# GDNF drives rapid tubule morphogenesis in a novel 3D *in vitro* model for ADPKD

**DOI:** 10.1242/jcs.249557

**Published:** 2020-07-16

**Authors:** Eryn E. Dixon, Demetrios S. Maxim, Victoria L. Halperin Kuhns, Allison C. Lane-Harris, Patricia Outeda, Andrew J. Ewald, Terry J. Watnick, Paul A. Welling, Owen M. Woodward

**Affiliations:** 1 University of Maryland School of Medicine, Department of Physiology, Baltimore, MD 21201, USA; 2 University of Maryland School of Medicine, Department of Medicine, Baltimore, MD 21201, USA; 3 Johns Hopkins University School of Medicine, Department of Cell Biology, Baltimore, MD 21205, USA; 4 Johns Hopkins University School of Medicine, Departments of Medicine and Physiology, Baltimore, MD 21205, USA

**Keywords:** 3D cell model, Epithelia, Kidney, Tubulogenesis, Collecting duct, Polycystic kidney disease

## Abstract

Cystogenesis is a morphological consequence of numerous genetic diseases of the epithelium. In the kidney, the pathogenic mechanisms underlying the program of altered cell and tubule morphology are obscured by secondary effects of cyst expansion. Here, we developed a new 3D tubuloid system to isolate the rapid changes in protein localization and gene expression that correlate with altered cell and tubule morphology during cyst initiation. Mouse renal tubule fragments were pulsed with a cell differentiation cocktail including glial-derived neurotrophic factor (GDNF) to yield collecting duct-like tubuloid structures with appropriate polarity, primary cilia, and gene expression. Using the 3D tubuloid model with an inducible *Pkd2* knockout system allowed the tracking of morphological, protein, and genetic changes during cyst formation. Within hours of inactivation of *Pkd2* and loss of polycystin-2, we observed significant progression in tubuloid to cyst morphology that correlated with 35 differentially expressed genes, many related to cell junctions, matrix interactions, and cell morphology previously implicated in cystogenesis.

This article has an associated First Person interview with the first author of the paper.

## Abstract (precis)

**Summary:** A new three-dimensional *in vitro* model drives tubule morphogenesis with the addition of GDNF and demonstrates methods for interrogating the initiation of ADPKD cystogenesis.

## INTRODUCTION

**Fig. 1.**
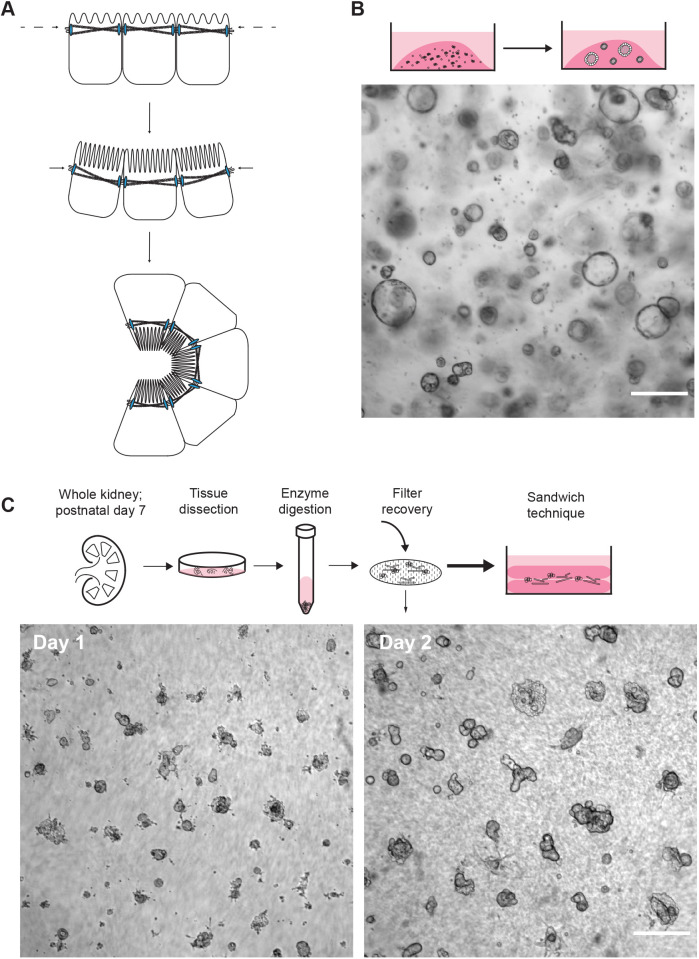
**Rationale for 3D culture of primary cells.** (A) Schematic representation of the change in morphology from 2D to 3D culture. (B) Immortalized single cells (*Pkd2^fl/fl^* *Pax8rtTA TetOCre Sv40*) are resuspended and cultured in Matrigel dots with renal growth factor medium. Spheroids develop from single cells and cellular aggregates. A representative bright-field image is shown. (C) Schematic representation of tubule fragment preparation from whole mouse kidney. Below, representative bright-field images show primary tubule fragments seeded between two layers of Matrigel on day 1 and day 2 of culture, demonstrating a succinct protocol for tubule development. Scale bars: 250 μm.

*In vitro* models of stem cell differentiation and kidney development have set the stage for expanding our understanding of nephrogenesis. Recognition that three-dimensional (3D) culture systems support the development of normal structure and physiology better than 2D non-polarized systems (Fig. 1A) has led to a paradigm shift in the field (Duval et al., 2017). Thus far, new 3D culture systems with embryonic or adult stem cells have led to advances in understanding diseases of the lung, intestines, brain, and now the kidney (Clevers, 2016; Takasato et al., 2016; Schutgens et al., 2019; Morizane et al., 2015). Optimized variations of 3D epithelial culture using induced pluripotent (iPSC) and adult (ASC) stem cells have led to novel kidney model systems that are able to recapitulate intermediate mesoderm, metanephric mesenchyme, ureteric epithelium, and proximal tubules (Freedman et al., 2015; Takasato et al., 2016; Schutgens et al., 2019). Further, introduction of specific mutations by CRISPR-Cas9 gene editing can be used to model human diseases of the kidney (Clevers, 2016; Freedman et al., 2015). These intricate models remain unmatched in recapitulating the complexity of the developing kidney. However, organoid complexity is not always a desirable feature, especially for studies focused on identifying and characterizing molecular pathways that drive tubulogenesis, epithelial cell differentiation, and complex disease processes, such as autosomal dominant polycystic kidney disease (ADPKD) (Clevers, 2016).

Roadblocks of current *in vitro* model systems fall into categories of cost, relevancy, and translational applications, which all stem from efforts to maintain epithelial-like structures and prevent them from reverting back to innate stemness (Dixon and Woodward, 2018). Despite the intricate characterization of these kidney organoids and personalization of the models to patients, the resulting structures demonstrate insufficient functional relevance and often rely on a nascent phenotype, which makes interpretation for late stage diseases, such as ADPKD, challenging (Forbes et al., 2018; Takasato et al., 2015). Recently, Schutgens et al. (2019) have expanded the niche of stem cell models by creating a tubuloid system that uses adult stem cells from human cortex biopsies to create epithelial structures. Unfortunately, the application of stem cell models to study genetic cystogenic mechanisms is limited, and these limitations are much the same as those of the foundational 3D *in vitro* models, which used immortalized cell lines and focused on spheroid development, including changes in number and size (Grantham et al., 1989; Mangoo-Karim et al., 1989).

Here, we describe a tubuloid model system that employs tubule fragments derived from whole murine postnatal kidneys. This approach preserves a progenitor cell population that can be manipulated to selectively drive the upregulation of epithelial differentiation and collecting duct associated signaling pathways. Unlike other segments, the differentiation, tubulogenesis, and extension of the Wolffian duct is largely driven by a single growth factor, glial-derived neurotrophic factor (GDNF) (Majumdar et al., 2003). Additionally, increased levels of GDNF expand populations of collecting duct progenitor cells (Li et al., 2019). The use of a primary cell 3D system driven to a desired tubule-like phenotype could be used to isolate changes in epithelial organization following acute experimental perturbations. Therefore, we designed a system that would be inexpensive, utilize commercially available reagents, allow for differentiation and experimental protocols to be completed in less than 2 weeks, and that would allow tracking and imaging of tubuloids *in situ* (Dixon and Woodward, 2018).

The significance of this new model development is driven by the lack of understanding of cystogenesis in tubular cystic diseases, including autosomal dominant (ADPKD) and recessive (ARPKD) polycystic kidney disease, and nephronophthisis. These cystic diseases share a phenotypic profile that includes presentation of collecting duct or medullary cysts (Hildebrandt and Zhou, 2007; Bergmann et al., 2018), supporting the need for development of a nonproximal *in vitro* model. Current models that employ knockout approaches of specific causal cystogenic genes, such as siRNAs or CRISPR, might mislead our understanding of initiating events in cystogenic mechanisms because they are not able to directly compare changes to epithelial cells before and after genetic perturbation (Cruz and Freedman, 2018; Freedman et al., 2015; Cruz et al., 2017). With multiphasic pathologies, the current *in vitro* model systems for cystogenic diseases represent a summary of epithelial changes rather than isolation of proximate changes to gene loss. Our new *in vitro* system presents a platform for investigating these cystic diseases in an isolated tubule-like structure.

The most prevalent of the aforementioned genetic epithelial cystic diseases is ADPKD (Igarashi and Somlo, 2002; Cornec-Le Gall et al., 2019). ADPKD is caused by loss-of-function mutations in *PKD1* and *PKD2*, which encode two transmembrane proteins, polycystin-1 (PC1) and polycystin-2 (PC2). The functions of the polycystin proteins in renal epithelial cells remain undefined, but they are hypothesized to form a channel complex with roles in cell migration and signaling (Hanaoka et al., 2000; Delmas et al., 2004; Su et al., 2018). The polycystins have been localized to the plasma membrane, cilia, endoplasmic reticulum, and cellular junctions (Yoder et al., 2002; Roitbak et al., 2004; Köttgen and Walz, 2005). Renal cystogenesis occurs when germline loss-of-function mutations in *PKD1* or *PKD2* are coupled with an additional somatic mutation in the *PKD* gene inherited from the unaffected parent (Watnick et al., 1998; Pei et al., 1999; Tan et al., 2018). When polycystin signaling falls below a critical threshold, mutant cells undergo morphological changes, increases in cell proliferation, changes in fluid secretion, and alterations in numerous signaling pathways (Gallagher et al., 2010; Pei, 2001; Ye and Grantham, 1993; Chapin and Caplan, 2010). As cysts clonally expand in the kidney, neighboring nephrons become damaged, resulting in compensatory hyperfiltration, eventually resulting in inflammation and fibrosis.

This complicated multiphasic pathology and poor understanding of the function of the polycystin proteins has left the pathological mechanisms of cystogenesis incompletely understood. Current stem cell based *in vitro* models used to study cystogenesis (Cruz et al., 2017; Freedman et al., 2015) in ADPKD focus on explaining proliferation, fluid accumulation, and changes in cell–matrix interactions, suffering the same restraints as older 3D *in vitro* models that used immortalized cell lines to focus on spheroid development and expansion (Grantham et al., 1989; Mangoo-Karim et al., 1989). The conclusions drawn from both immortalized and stem cell PKD models tend to conflate potential downstream repercussions of polycystin loss, such as proliferation and fluid secretion, with causal factors in cystogenesis. Current models do not provide the possibility to observe morphological changes resulting from acute polycystin loss in an intact tubuloid or tubule structure (Grantham et al., 1989; Mangoo-Karim et al., 1989; Cruz et al., 2017).

Here, we present a new tubuloid model system that is designed to overcome many of the major current roadblocks to our understanding of the initiating steps in epithelial morphological change and cystogenesis. We used mouse renal tubule fragments containing *Six2*-positive progenitor cells pulsed with GDNF to yield collecting-duct-like tubuloid structures. For proof of principle, we used tubuloids generated from an inducible *Pkd2* knockout system (*Pkd2^fl/fl^ Pax8rtTA TetOCre +mTmG*), demonstrating the ability to track the morphological, protein, and genetic changes that correlate with observable and quantifiable cyst formation. This model system is well suited for studying the proximate pathological mechanisms of cystogenesis resulting from genetic cystic diseases; mechanisms that might be amenable to therapeutic targeting.

## RESULTS

### Novel development of tubules and differentiated structures via primary cell culture in a Matrigel sandwich

Two-dimensional *in vitro* models have provided critical insight into the cell biology of epithelial mechanisms of proliferation and fluid secretion, but there is a limit to the relevance and translation of 2D cultures to physiological mechanisms. This disconnect is in part due to the dramatic change in morphometry and organization of epithelial cells on flat, stiff substrates (Fig. 1A). Therefore, we opted for a 3D system employing Matrigel to establish a synthetic basement membrane matrix. The rationale behind the development of this system was to drive and maintain the formation of specific tubule structures through manipulation of the basement membrane and growth factors. To begin, we resuspended single cells and cell aggregates from a renal epithelial immortalized cell line (*Pkd2^fl/fl^ Pax8rtTA TetOCre Sv40*; see Materials and Methods) into Matrigel droplets (Fig. 1B) (Sato and Clevers, 2013). After exposure to kidney specific growth factor enriched medium, cells proliferated and exclusively expanded into fluid-filled spheroids – the apparent default structure for epithelial cells in 3D culture, independent of the polycystin status (Benton et al., 2014; Dutta et al., 2017). Critically, we were unable to generate tubule-shaped structures. We hypothesized that morphological changes precede alterations to proliferation and secretion after polycystin loss. Lack of tubular structures with the immortalized renal epithelial cell lines, before inactivation of the *PKD* genes, prevented the testing of this hypothesis.

Based on this observation, we made two critical changes to the plating protocol to aid in the development of tubules. Firstly, primary cell material, isolated from a postnatal day 7 mouse kidney, was used instead of an immortalized cell line. Secondly, tubule fragments, isolated by digestion with a collagenase solution, were seeded in between two layers of polymerized Matrigel, a method known as the sandwich technique (Fig. 1C). This culture technique allowed us to create a single focal plane of structures, which was helpful for tracking and imaging in a single field of view. Additionally, the seam in the matrix appeared to increase diffusion of the culture medium and increase exposure of the plated structures to nutrients and growth factors, allowing rapid tubule formation within 48 h of culture (Fig. 1C).

**Fig. 2.**
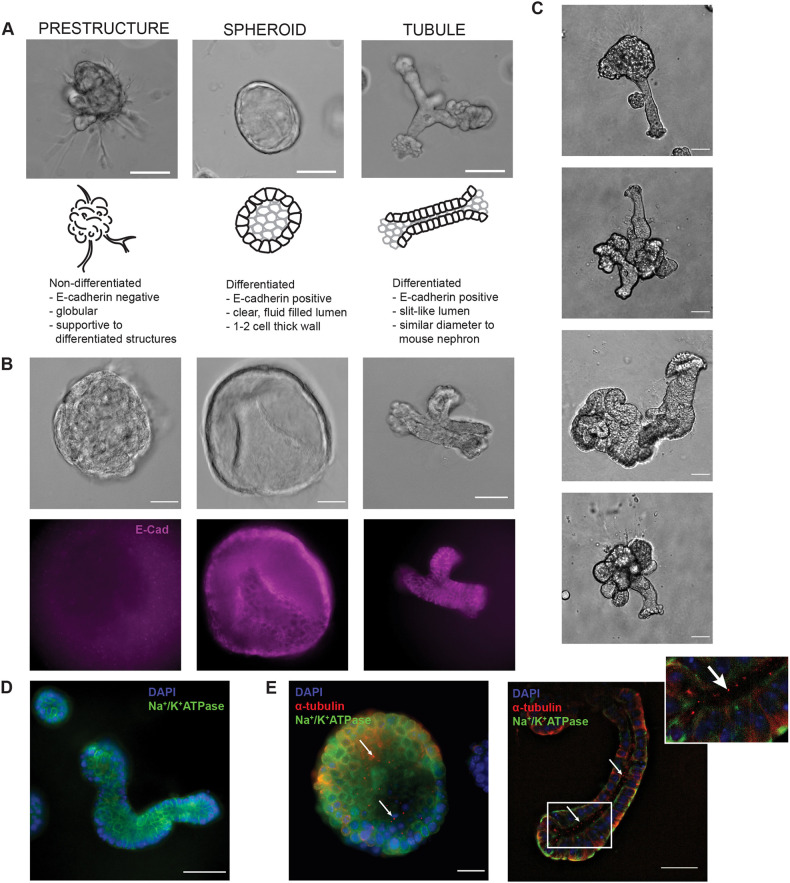
**Characterization of unique 3D structures.** (A) Representative bright-field image, schematic, and description for each class (prestructure, spheroid, and tubule) of organoid structures. Scale bars: 100 μm. (B) Bright-field images and corresponding immunofluorescence staining for E-cadherin (purple) in non-differentiated (E-cadherin negative; prestructures) and differentiated (E-cadherin positive; spheroids and tubules) structures. Scale bars: 50 μm. (C) Representative bright-field images of tubule development in the culture model. Tubules are often part of more complex 3D structures, including the globular, supportive prestructures. Scale bars: 25 μm. (D) Differentiated tubule structures demonstrate basolateral localization of Na^+^/K^+^-ATPase (green). Nuclei are stained with DAPI (blue). Scale bar: 50 μm. (E) Spheroids and tubules additionally demonstrate primary cilia, as indicated by acetyl-α-tubulin (red) puncta on the apical membrane. Basolateral Na^+^/K^+^-ATPase (green) and nuclear DAPI staining (blue) are also shown. Arrows indicate cilia, positively stained by acetyl-α-tubulin. Scale bars: 25 μm.

The combination of primary cell fragments and Matrigel plating strategy with a renal specific growth factor medium resulted in the development of two categories of 3D structures: prestructures, and tubuloids (either spheroids or tubules; Fig. 2A). The first structure class, prestructures, were globular and categorized as being non-differentiated (E-cadherin negative; Fig. 2B). These were often found supporting other differentiated (E-cadherin positive) tubuloid structures (Fig. 2B,C). Differentiated tubuloids were further segregated by morphology into spheroids, defined by a circular, clear lumen and a spheroid wall that was 1–2 cells wide, or tubules, defined as a structure with a slit-like lumen and approximately 50 μm in diameter, mimicking the approximate size of a mouse nephron (Fig. 2C). Both spheroids and tubules demonstrated the expected polarization of characteristic markers, including the basolateral transporter Na^+^/K^+^-ATPase and the apical primary cilia marker acetyl-α-tubulin (Fig. 2D,E).

### Using GDNF to promote epithelial development and drive formation of collecting-duct-like tubuloids

**Fig. 3.**
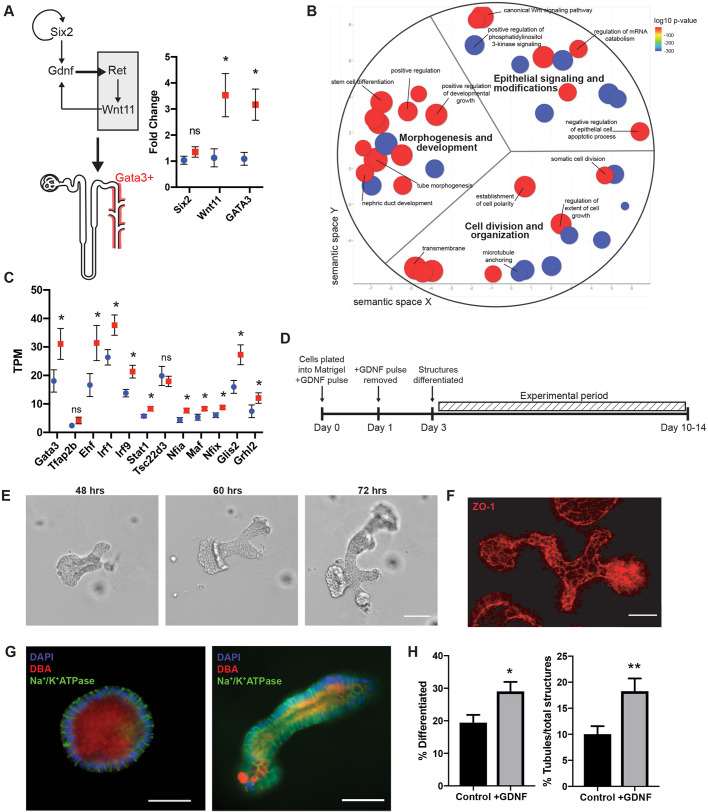
**Effect of GDNF on organoid differentiation.** (A) Schematic of the GDNF–Ret signaling axis that drives formation of the collecting duct system. Relative mRNA fold change between control (*n*=4 cultures for each group, blue circle) and GDNF pulse (*n*=4 cultures for each group, red square) organoids for the stem cell marker, *Six2* (*P*=0.2854, two-tailed Student's *t*-test), and the downstream GDNF effectors *Wnt11* and *Gata3* (*Wnt 11*, *P*=0.0367; *Gata3*, *P*=0.0184; two-tailed Student's *t*-test). Data shown are mean±s.e.m. (B) ReviGO representation of significantly changed (nominal *P*<0.05) GO terms in GDNF pulsed organoids [*n*=4 cultures, dot size=log_10_ frequency (percentage of genes annotated) with that GO term (Supek et al., 2011), dot color=log_10_ *P*-value]. (C) Principal cell-specific transcription factors are significantly upregulated in GDNF pulsed tubuloids (*n*=4 cultures for each group, red square) when compared to controls (*n*=4 cultures for each group, blue circle). Significant upregulation of *Gata3* (*P*=0.0499), *Ehf* (*P*=0.0455), *Irf1*(*P*=0.0225), *Irf9* (*P*=0.0132), *Stat1* (*P*=0.0220), *Nfia* (*P*=0.0168), *Maf* (*P*=0.0239), *Nfix* (*P*=0.0171), *Glis2* (*P*=0.0175), and *Grhl2* (*P*=0.0789) determined by one-tailed Student's *t*-test. Data shown are mean±s.e.m. (D) Schematic experimental paradigm for the GDNF pulse. (E) Morphometry tracking of tubuloids. Representative bright-field images of tracked tubule bifurcation events from 48 h to 72 h post-plating. Scale bar: 100 μm. (F) 3D SIM reconstruction of ZO-1 (red) to demonstrate junctional organization in tubuloid structures. Scale bars: 25 μm. (G) Immunofluorescence images of a differentiated spheroid and tubule positive for collecting duct marker, DBA (red) with basolateral Na^+^/K^+^-ATPase (green) and DAPI (blue). Scale bars: 50 μm. (H) Quantification of structure classification in bright-field images taken with 4× objective magnification reveals that the addition of GDNF yields more differentiated structures (*P*=0.0102; two-tailed Student's *t*-test) that are tubules (*P*=0.0091; two-tailed Student's *t*-test.) when compared to control. Data shown are mean±s.e.m. *n*=574 structures, three tubuloid preparations and three mice for control and *n*=526 structures, three tubuloid preparations and three mice for +GDNF. **P≤0.01; *P≤0.05; ns, not significant.

We sought to increase the yield of tubules by taking advantage of the inherent plasticity of the system and presence of progenitor cells found in the still developing postnatal kidney, as evidenced by the expression of *Six2* [1.34 mRNA transcripts per million, *n*=4 independent tubuloid populations (data not shown); adult mouse kidney does not express *Six2* (Maglott et al., 2005)]. We treated the cultures with a 24 h pulse of exogenous glial-derived neurotrophic factor (GDNF) with continuous exposure to hepatocyte (HGF), epidermal (EGF), and fibroblast (bFGF, also known as FGF2) growth factors. GDNF is responsible for extension of the Wolffian Duct into the metanephric mesenchyme, ultimately making the GATA3-positive collecting duct system (Fig. 3A), acting through the Ret receptor and Wnt11 signaling (Majumdar et al., 2003). Reverse transcription quantitative PCR (RT-qPCR) revealed that downstream indicators of GDNF–Ret action, including *Wnt11* and *Gata3*, were significantly increased following a 24 h pulse of GDNF when compared to levels in controls (Fig. 3A). Next, we extracted structures with and without exposure to GDNF (*n*=4 cultures of each), isolated RNA, and performed RNA-Seq analysis to observe the changes in gene expression influenced by the GDNF pulse. Gene ontology (GO) term analysis demonstrated that genes critical in morphogenesis and epithelial differentiation had significantly changed expression after GDNF pulse as compared to controls (Fig. 3B; Tables S1, S2). We hypothesized that the addition of a GDNF pulse would drive a collecting duct phenotype in our tubuloid cultures. However, tubuloids did not seem to exhibit mature collecting duct markers, such as *Fxyd4*,* Aqp2*, and *Avpr2* (Chen et al., 2017). Next, we sought to establish whether the tubuloids might be collecting duct precursors by determining whether there was upregulation of collecting duct-specific transcription factors following GDNF pulse. Indeed, we found that 10 out of 12 principal cell-specific transcription factors were upregulated in GDNF tubuloids (Fig. 3C; Table S3; *n*=4, one-tailed Student's *t*-test, *P*≤0.05) (Chen et al., 2017). Activation of these gene networks by the GDNF pulse appears to significantly speed up the process of structural differentiation, resulting in differentiated tubuloid structures in 3 days (Fig. 3D). Consistent with the gene expression analysis, we also observed significant increases in total number of differentiated structures (two-tailed Student's *t*-test, *P*=0.0102; *n*=574 control structures, three tubuloid preparations and three mice; *n*=526 structures, three tubuloid preparations and three mice for +GDNF) and proportion of differentiated tubuloids that are the desired tubule shape (two-tailed Student's *t*-test, *P*=0.0091; *n*=574 structures and three tubuloid preparations and three mice for control; *n*=526 structures and three tubuloid preparations and three mice for +GDNF) (Fig. 3H). Differentiated structures were considered to be either spheroids or tubules according to the descriptions in Fig. 2A (see Materials and Methods for more details). Finally, we also evaluated the effects of GDNF exposure duration and found that durations longer than the 24 h pulse, including continuous exposure, did not produce any difference in the yield of tubules (*P*=0.2037). Employment of an automated stage setup for image acquisition allowed tracking of individual structures through development in a similar plane of focus (Fig. 3E). Using this method, we tracked structures following the GDNF pulse and found they demonstrated tightly organized junctions (as indicated by expression of tight junction protein ZO-1) and stained positively for the collecting duct and principal cell marker DBA (*Dolichos biflorus* agglutinin; Fig. 3F,G). DBA-positive staining of differentiated tubuloids in our system reinforced the upregulation of principal cell-specific transcription factors (Fig. 3C) (Chen et al., 2017; Labarca et al., 2015; Murata et al., 1983).

### Tracking and assessing polycystin inactivation in doxycycline-treated organoids

**Fig. 4.**
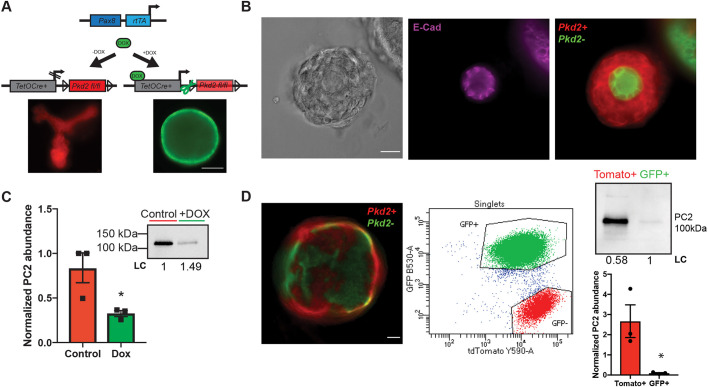
**Addition of doxycycline results in inactivation of *Pkd2* in 3D culture.** (A) Schematic of genetics for inducible Cre model system used for *Pkd2* inactivation. Representative images show structures expressing the mTmG reporter with (right, green) and without (left, red) addition of doxycycline (DOX) to induce *Pkd2* inactivation. Scale bar: 100 μm. (B) Bright-field and immunofluorescence images demonstrating that epithelial cells (E-cadherin positive, purple) respond to doxycycline (*Pkd2+*, red; *Pkd2−*, green). Scale bar: 25 μm. (C) Western blot and quantification for PC2 abundance, normalized to total loaded protein (LC). Data shown are mean±s.e.m. *n*=3 tubuloid preparations from individual animals. *P*=0.0420 (two-tailed Student's *t*-test). (D) Fluorescence image showing heterogenous inactivation of *Pkd2^fl/fl^* via the double membrane reporter mTmG, with *Pkd2*+ cells shown in red and *Pkd2*− cells in green. Fluorescence activated single cell sorting (*n*=1.58×10^6^ cells for GFP+ and 213,000 cells for GFP−) of *Pkd2^fl/fl^* *Pax8rtTA TetOCre +mTmG* primary cells following treatment with doxycycline. Western blotting confirms a significant decrease in PC2 abundance in green fluorescing cells, normalized to total loaded protein (LC). Data shown are mean±s.e.m. *P*=0.0336 (two-tailed Student's *t*-test). Scale bar: 50 μm. *P≤0.05.

To test the model as a potential assay for studying cystogenesis as caused by ADPKD, kidney cells from an inducible genetic mouse model of *Pkd2* inactivation (*Pkd2^fl/fl^ Pax8rtTA TetOCre +mTmG*) were employed (cells from a *Pkd1^fl/fl^* *Pax8rtTA TetOCre +mTmG* mouse models also work; Fig. S1). In this system, a tetracycline transactivator is driven by a renal-epithelia-specific *Pax8* promoter. When doxycycline, a tetracycline derivative, is added to the culture, a responsive Cre recombinase cleaves the floxed *Pkd2* gene and results in a change from red to green fluorescence due to a double fluorescent membrane reporter (mTmG) (Fig. 4A). The action of the Cre recombinase was restricted to epithelial (E-cadherin-positive) cells in culture due to the *Pax8* promoter (Fig. 4B), with prestructures not responding to the addition of doxycycline. When tubuloids were recovered from the Matrigel sandwich following doxycycline treatment, there was a significant decrease in PC2 protein abundance as assessed by western blotting (Fig. 4C; Fig. S2A–C; two-tailed Student's *t*-test, *P*=0.0420; *n*=3 tubuloid preparations from individual animals). However, PC2 was still detectable, and red cells (presumably wild type) were observed in differentiated structures (Fig. 4C,D). To confirm the correlation between Cre activation, mTmG conversion, *Pkd2* inactivation, and loss of PC2, we used fluorescent activated cell sorting. We found the abundance of PC2 in green fluorescing cells was significantly less than in red fluorescing cells, establishing our use of the color change as a marker for *Pkd2* expression (Fig. 4D; Fig. S2D–F; two-tailed Student's *t*-test, *P*=0.0336; *n*=1.58×10^6^ cells for GFP+ and 213,000 cells for GFP−).

**Fig. 5.**
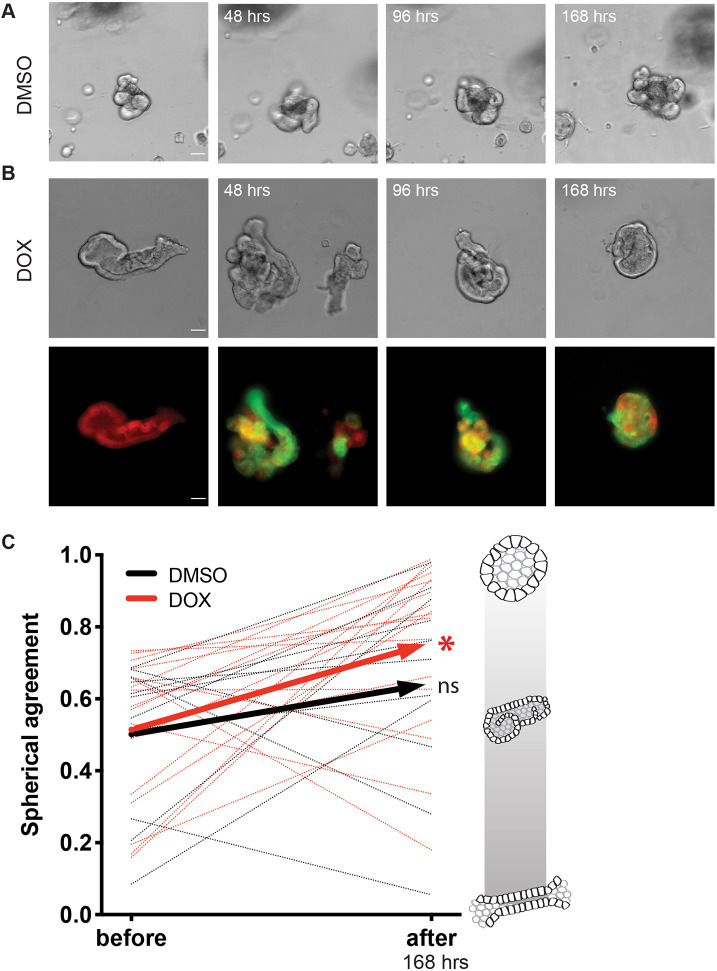
**Loss of Pkd2 changes tubuloid morphology.** (A) Bright-field images of a tracked control tubuloid before and after the addition of DMSO at 48, 96, and 168 h timepoints. (B) Bright-field tracking with corresponding fluorescence of +mTmG reporter following morphometrical changes before addition of doxycycline and at 48, 96, and 168 h timepoints post doxycycline (DOX) treatment. (C) Tubuloids treated with doxycycline demonstrate a significant (*P≤0.01) increase in spherical agreement, or spheroid likeness, when comparing morphometry before and after inactivation of *Pkd2* (*n*=20 differentiated structures, red dotted lines; two-tailed *P*=0.0072, before and after paired *t*-test). Control structures (DMSO) do not significantly change over the same 168 h timecourse (*n*=20 differentiated structures, black dotted lines; two-tailed *P*=0.1746, before and after paired *t*-test). Average of doxycycline-treated (red) and DMSO-treated (black) tubules change in spherical agreement represented by solid lines. Scale of spherical agreement is shown with schematic renderings of tubule (0) to spheroid (1). Scale bar: 50 μm.

We tracked individual tubuloid structures after the inactivation of *Pkd2* and compared them to DMSO-treated or control structures following the same timeline (Fig. 5A,B). We described and quantified morphological changes of individual structures before and after treatment with doxycycline or DMSO by applying a metric of spherical agreement, which assesses whether individual structures become more spheroid and cyst-like (Fig. 5C). Spherical agreement was determined by finding the difference between spherical-like area of each structure (area=2πr^2^) and the actual area (see more detail in Materials and Methods). We found the individually tracked tubuloids exposed to the doxycycline treatment demonstrated an increase in spherical agreement, becoming more cystic in 72 h (*n*=20 differentiated structures, *P*=0.0072, before and after paired *t*-test) (Fig. 5C). Structures treated with DMSO did not significantly change over the same 72 h period (*n*=20 differentiated structures, *P*=0.1746, before and after paired *t*-test). Interestingly, we did not see a doxycycline-dependent change in tubuloid structure size (*P*=0.1255; data not shown), suggesting that the morphological changes observed with doxycycline treatment were independent and precede expected increases in proliferation rate with polycystin loss. We additionally evaluated expression of 11 known epithelial proliferation marker genes and did not find any significant changes following doxycycline treatment when compared to expression in DMSO-treated control tubuloids (Fig. S3, Table S4) (Shehata et al., 2018; Combes et al., 2019; Dudderidge et al., 2005; Yamaguchi et al., 2006). These results demonstrated that the inactivation of *Pkd2* drives a morphological, rather than a proliferative, change in this tubuloid model system at the time points observed.

### The 3D tubule system integrates relevant PKD matrix genes

**Fig. 6.**
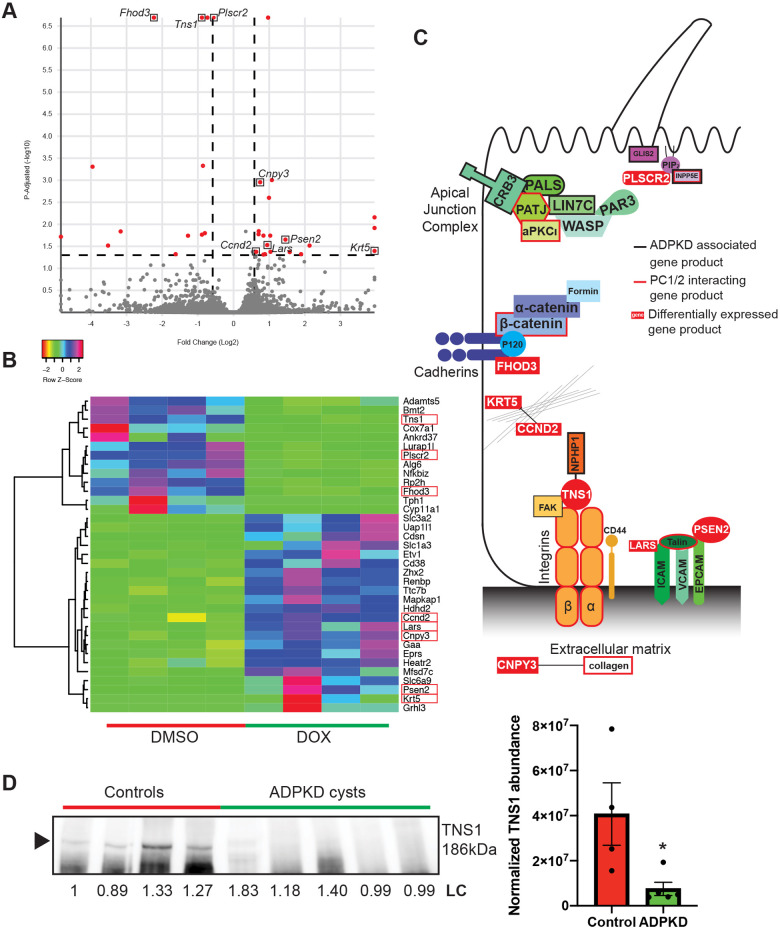
**Differentially expressed ADPKD related genes.** (A) Volcano plot representing differentially expressed genes (red) in doxycycline-treated organoids (*Pkd2-*) with adjusted *P*≤0.05 and fold change ≥1.5 (*n*=4 cultures for each group). (B) Heatmap showing directionality of expression for significantly (adjusted *P*≤0.05) altered genes in doxycycline-treated organoids compared to controls. Genes involved in junctions and the matrix are highlighted with red boxes. (C) Schematic of proteins that have been reported as ADPKD associated (black outline) or reported/predicted to interact with PC1 or PC2 (red outline). Proteins encoded by differentially expressed genes are shown in relationship to the other complexes and highlighted by white text in a red box. (D) Western blotting reveals a significant decrease in tensin-1 (TNS1) in human male ADPKD cysts (*n*=5 cysts) compared to normal human male kidneys (*n*=4 control samples), normalized to total loaded protein (LC). Arrowhead indicates the TNS1 band. Data shown are mean±s.e.m. *P*=0.0340 (two-tailed Student's *t*-test). *P≤0.05.

To better asses the utility of the 3D tubule system, we next characterized gene expression changes after *Pkd2* knockout. We recovered tubuloid structures 2 days following treatments with either doxycycline or DMSO (*n*=4 cultures for each group), isolated RNA, and performed an RNA-Seq analysis. Surprisingly, we found only a small number of genes (35) had differentially altered expression following treatment with doxycycline and loss of *Pkd2* (adjusted *P*=0.05; 1.5-fold change up or down) (Fig. 6A,B; Table S5). Among these 35 differentially expressed genes were many whose protein products localize and function in the junctions and extracellular matrix of epithelial cells (red boxes, Fig. 6B). Many of the proteins associated with these differentially expressed genes have been implicated in cystic phenotypes, or as potential interactors with the polycystins (Fig. 6C; Table S6). One such gene is *Tns1*, which codes for the integrin adapter protein tensin-1 (Table S6). Previously, it has been shown that knock out of *Tns1* in mice produces cysts in the kidney (Lo et al., 1997), and tensin-1 interacts with nephrocystin-1 (NPHP1) (Benzing et al., 2001). We explored alterations in tensin-1 protein abundance in human kidney cysts from ADPKD patients and found an almost complete depletion of the tensin-1 protein in the cystic tissue as compared to levels in normal human kidney tissue (Fig. 6D; Fig. S4; *n*=4 control samples and *n*=5 ADPKD cysts). Taken together, these findings demonstrate the power of this system to better understand both the normal role of the polycystins in tubulogenesis, and their pathogenic role in cystogenesis. Furthermore, this proof of principle study strongly suggests that this tubuloid system could similarly provide insights into other genetic diseases of the epithelium.

## DISCUSSION

The development of epithelial tubules is driven by mechanisms dependent on the polarity of cells (Bernascone et al., 2017). This orientation, and therefore promotion of relevant epithelial organization *in vitro*, is dependent on the interactions between neighboring cells as well as the extracellular matrix (Iruela-Arispe and Beitel, 2013; Bernascone et al., 2017). These adhesive interactions drive the establishment of polarity and eventually the organization of the lumen; the cell division machinery continues to integrate these signals to maintain appropriate orientation of the structure during proliferation (Iruela-Arispe and Beitel, 2013). The players that define these interactions include proteins and complexes that interact with the junctions and integrins of epithelial cells. The formation of cysts in renal tubules, as occurs in ADPKD, ARPKD, and nephronophthisis, is a pathological disruption of tubulogenesis, and it has been demonstrated previously that changes to planar cell polarity result in dilation of tubules (Bernascone et al., 2017). To understand precisely the proteins and genes essential for maintenance of tubules, as well as the disruption of tubulogenesis associated with cystogenic diseases, there needed to be a new 3D culture system that could mimic the interaction between tubules, extracellular matrix, and perturbations occurring after acute genetic alterations.

Here, we have focused on the most common cystic kidney disease, ADPKD, to illustrate the benefits of our new tubuloid system. Previously, the polycystins have been implicated in epithelial differentiation and establishment of tubules (Boletta, 2003; Boletta et al., 2000; Grimm et al., 2006). Furthermore, polycystins have a noted relationship with the microenvironment (Cruz et al., 2017; Drummond, 2011), but there is not a current model system to determine the immediacy of these changes in response to the loss of gene function in relevant epithelial structures. Although decades of work have led to exciting advances in our understanding of disease pathology and the first FDA-approved treatments, the initiating mechanisms underlying progressive cystogenesis remain undefined in renal epithelial cells (Dixon and Woodward, 2018; Torres and Harris, 2019; Brill and Ehrlich, 2020). Dissecting the complicated pathogenesis of PKD into distinct phases in an isolated tubuloid structure might allow more accurate targeting of therapeutic development and was an obvious unmet need that drove our development of a new model system.

Loss-of-function mutations in either *PKD1* or *PKD2* are associated with downstream changes, including increased proliferation, changes in apicobasolateral organization, alterations in cell–matrix interactions, and fluid secretion and accumulation (Terryn et al., 2011; Drummond, 2011; Cornec-Le Gall et al., 2019). Unfortunately, understanding how the many different pathways and morphological changes are related has remained intractable (Antignac et al., 2015; Torres and Harris, 2014; Bergmann et al., 2018). Animal models that produce the most physiologically relevant tools for recapitulating ADPKD and other cystic diseases (Dukes et al., 2011; Arthur, 2000) reflect this complexity, making teasing out temporal sequence or distinct linearity in progression difficult. *In vitro* models, if not built for a specific question, also present levels of complexity that obscure important initiating steps in cystogenesis, either from clonal variation, inappropriate substrates, or heterogenous segmental origin. Specifically, recent advancements in iPSC-derived kidney organoids often fail to model pathology of human disease because of their early developmental kidney phenotypes (Takasato et al., 2015; Forbes et al., 2018). Additionally, previously published renal organoid systems require weeks to display renal cell phenotypes (Morizane et al., 2015; Takasato et al., 2016). The extended culture times necessary for these experiments require replacement of the synthetic basement membrane or matrix for structures in 3D culture to establish a mature phenotype (Freedman et al., 2015), making tracking structures through time impossible. Ultimately, these limitations have resulted in a failure to fully understand the immediate downstream consequences of the loss of *PKD* gene expression.

To overcome these limitations, we have designed a new *in vitro* model system that improves on previous 3D *in vitro* models to specifically address the mechanisms of cystogenesis (Thatava et al., 2011; Freedman et al., 2013; Hofherr and Köttgen, 2013; Cruz et al., 2017). Our model system makes four critical improvements: (1) the use of primary mouse tubule fragments from whole kidney, (2) application of a Matrigel sandwich plating method to enhance tubuloid formation and visualization, (3) addition of glial-derived neurotrophic factor for rapid differentiation of tubuloids with upregulated collecting duct associated signaling pathways, and (4) the use of material from any extant mouse model, including those engineered with inducible technology. These innovations pragmatically increase experimental efficiencies for studying the key elements in cystogenesis: allowing tracking of morphology, protein abundance, and gene expression. First, we used primary tubule fragments as our starting material, which differentiate into mature tubuloids in ∼3 days. This allows further genetic inactivation and tracking for up to 14 days in the original matrix. Second, the use of a sandwich plating method, providing tubuloids with increased access to nutrients and oxygen, rapidly sped up tubuloid growth. This technique also positions the structures in a similar focal plane in the matrix, allowing for better visualization as well as tracking and imaging of the tubuloid structures. A third innovation was to drive the development of tubuloids towards a discrete segmental phenotype. Many of the stem cell-derived organoids mentioned previously contain various nephron segments and cell types (Takasato et al., 2015). This complexity, however, can confound the interpretation of experimental results. Thus, we used a GDNF pulse, to speed the tubuloid development and to drive the upregulation of collecting duct associated transcription factors in tubuloids. In development, GDNF is a key factor in the initiation of the Wolffian duct, which eventually becomes the collecting duct (Majumdar et al., 2003). As expected, expression of key players (*Wnt11*, *Gata3*) in the GDNF signaling pathway was significantly increased in our culture system following the exogenous pulse of GDNF (Majumdar et al., 2003; Grote et al., 2006). Gene expression pathways involved in the development of the nephric duct, as well as in the differentiation of the resident stem cell population and negative regulation of epithelial cell apoptotic processes, were all significantly upregulated following a pulse of GDNF (Fig. 3B). We found that the resulting tubuloids expressed collecting duct and principal cell marker (DBA), and lacked proximal markers [LTL, ABCG2 (data not shown)] (Patel and Dressler, 2013). We were unable to more specifically define subpopulations of tubuloids by protein expression and localization due to the inability of some antibodies for mature segment markers to penetrate the Matrigel sandwich for immunofluorescence (e.g. AQP2, NKCC1, V-ATPase). Careful gene expression analysis, unencumbered by the Matrigel, with further exploration of the growth factor cocktail components and exposure, as well as implementation of additional differentiation methods, might provide critical insight into producing a more fully characterized tubuloid segmental phenotype.

Application of this new system to study acute changes in cell biology following the temporally controlled inactivation of *Pkd2* and loss of PC2 protein provided significant insight into the initiating steps of cystogenesis. We employed tubule fragments from mice with a floxed *Pkd2* gene, inducible Cre, and mTmG reporter system. We took advantage of the ability to track structures by characterizing morphological, protein, and gene expression changes after the inactivation of *Pkd2*. The development of our spherical agreement analysis for morphometry presented a quantitative method to assess acute changes in tubuloid structure following *Pkd2* inactivation. This metric provided critical proof of principle that the model could produce pathogenic cysts, not just nonpathogenic spheroids. This analysis also provided evidence that the loss of *Pkd2* did not accelerate the rate of tubuloid growth, but only altered the morphology, demonstrating a distinct separation in morphological changes from the well characterized and apparent subsequent increases in proliferation (Hanaoka and Guggino, 2000; Grimm et al., 2006). We took advantage of the discrete morphological changes to learn more about the genes responsible for the initiation of cystogenesis. Interestingly, RNA-Seq analysis of differential message levels after *Pkd2* inactivation revealed changes in epithelial pathways that alter matrix and junctional genes that have been previously associated or implicated with the polycystins or cystic disease (Fig. 6C). Of note was the small number of genes with altered expression after doxycycline treatment and *Pkd2* loss, especially in contrast to the hundreds of differentially expressed genes following our GDNF pulse. It is possible we captured a stage of alteration after *Pkd2* loss that was restricted to the initiating stages of cystogenesis, where gene expression of only a small subset of key structural proteins are altered. We hypothesize that these morphological changes in turn will activate pathways that will increase cell proliferation and alter cell metabolism, cumulative changes captured in previous gene expression studies *in vivo* and other 2D cell models (Podrini et al., 2018; Menezes et al., 2012; de Almeida et al., 2016). The probable dissection of temporal stages in cystogenesis was the impetus for development of this new tubuloid system, and as demonstrated for ADPKD, the system might reveal key insights for other renal cystic diseases as well.

To further validate our approach and the findings from the RNA-Seq analysis of this new *in vitro* model, we investigated the changes in protein abundance of tensin-1, a gene significantly downregulated (*Tns1*, adjusted *P*=0.0000213), in human tissue. TNS1 was implicated in cystic kidney diseases by TNS1 knockout mouse models that developed small, but significant, cortical and medullary cysts, ultimately leading to death from renal failure (Lo et al., 1997; Wu et al., 2019). More recently, *TNS1* knockout MDCK cells have been observed to form multiluminal spheroids in 3D culture displaying alteration in morphology consistent with early stages of cystogenesis (Wu et al., 2019). Our results demonstrate that tensin-1 is significantly decreased in human APDKD tissue when compared to levels in normal human kidney. This is the first report of tensin-1 changes in human ADPKD and is critical evidence for the validation of our system in identifying potential targets for the initiation of cystogenesis. Interestingly, *TNS1* has previously been shown to interact with *NPHP1*, a causal gene underlying a second cystic renal disease, nephronophthisis (Benzing et al., 2001). Taken together, the data presented here suggests that the initiating events in cystogenesis are dominated by changes in how the cell interacts with other cells and the local environment, and that there might be some universality to these initiating steps across all genetic cystic diseases. These alterations would then progressively lead to secondary effects, like the clonal proliferation and fluid secretion, accompanied by disease specific pathogenesis leading to the observed constellation of emergent phenotypes in various genetic cystic diseases.

## MATERIALS AND METHODS

### Mice

The inducible Cre mice (*Pkd2^fl/fl^*, *Pax8rtTA*, *TetOCre*, *+mTmG*) were received from the Baltimore PKD Research and Clinical Core Center. Animal studies were performed in adherence to the NIH Guide for the Care and Use of Laboratory Animals and approved by the University of Maryland School of Medicine Institutional Animal Care and Use Committee. Mice were housed in groups of two to five per cage on a 12:12 h light/dark cycle with lights on at 6 a.m. The Baltimore PKD Research and Clinical Core Center genotyped the mice using primers for *Pkd2^fl/fl^* (forward, 5′-CCTTTCCTCTGTGTTCTGGGGAG-3′; reverse, 5′-GTTTGATGCTTAGCAGATGATGGC-3′), *Pax8* (forward, 5′-CCATGTCTAGACTGGACAAGA-3′; reverse, 5′-CTCCAGGCCACATATGATTAG-3′), and *Cre* (forward, 5′-ATTGCTGTCACTTGGTCGTGGC-3′; reverse, 5′-GGAAAATGCTTCTGTCCGTTTGC-3′) and standard thermocycling protocols.

### Immortalized cell spheroid culture

Immortalized single-cell preparations from *Pkd2^fl/fl^* *Pax8rtTA TetOCre Sv40* were generously provided by the Baltimore PKD Research and Clinical Core Center. These cells were cultured on collagen-coated transwells with 0.4 μm pores (Corning 3491) at 33°C. The cells were propagated at this temperature until 5 days past confluency and fed with the Baltimore PKD renal epithelia cell medium (REC): a 1:1 mixture of RenaLife Complete Medium (Lifeline Cell Technology LL-0025) and Advanced MEM medium (Fisher Scientific #12492) with 5% FBS (ThermoFisher #26140-079), 2.2% penicillin-streptomycin (Fisher Scientific #30-002-Cl), 0.6% L-alanyl-glutamine (Gemini Bio-products #400-106), and 0.03% gentamicin (Quality Biological #120-098-661). For propagation, 10 ng/ml interferon-gamma (CST 39127) was also added to the medium when cultured at 33°C. Polarized plates were then moved to 37°C and replaced with REC medium without interferon-gamma. For 3D culture of immortalized cells, six-well culture plates (Corning 3516) were prewarmed at 37°C overnight before plating. On the day of seeding (typically 2 days after transition to 37°C), cells were detached from the transwell using a cell scraper then pelleted using centrifugation. The medium from the pellet was aspirated and then the pellet was resuspended in growth factor reduced Matrigel (Corning 354230). This Matrigel-cell suspension was then plated across the prewarmed plate in 8–10 droplets per well. For droplet polymerization, the plate was left at room temperature to set for 10 min and then inverted to incubate for 20 min at 37°C. Following these incubations, the culture plate was righted, REC medium was added, and plates were incubated at 37°C for the remainder of the experiment. Spheroids developed in ∼3 days after plating.

### Primary tubule fragmentation

Kidneys were removed from anesthetized (with isoflurane >4.5%) postnatal day 4–7 mice and kept on ice in DMEM/F12 (Life Technologies 10565-018) medium for transfer into sterile culture conditions. Collagenase A solution was prepared by combining the following: 9.5 ml DMEM/F12, 5 μl gentamicin (50 mg/ml, Life Technologies 15750-060), 5 μl insulin (10 mg/ml, Sigma 19278), 0.5 ml FBS (Sigma F0296), 115 μl DNAse I (2 U/ml, Sigma D4263), and 200 μl collagenase A (0.1 g/ml, Sigma C2139). Kidneys were dissected into 1 mm^3^ sections and washed with 2 ml of collagenase A solution. Primary tissue sections were transferred into a 15 ml falcon tube treated with 2.64% BSA (Sigma A9576) in 1×d-PBS (Gibco 10010-023) with a final volume of 2 ml. Dissected preparations were then incubated for 1 h at 37°C in a water bath, shaking at 125 rpm. Periodically, tissue preparations were mechanically disrupted with a 2.64% BSA treated 1 ml pipette tip. Following the incubation, 8 ml of cold DMEM/F12 was added to inactivate the enzymatic reaction. The inactivated suspension was filtered through a Pluristrainer (20 μm, Pluriselect 43-50020-03) to capture larger, tubule fragments and exclude single cells. The Pluristrainer was washed with 10 ml DMEM//F12 and transferred into a 2.64% BSA-coated falcon tube. The preparations were centrifuged for 3 min at 500 ***g*** and the supernatant was aspirated to remove any residual collagenase A solution. The remaining pellet of epithelial tubule fragments was resuspended in freezing medium (90% FBS, 10% DMSO) and stored at −80°C.

### Culture and differentiation

Aliquots of basement membrane construct, growth factor reduced Matrigel (Corning 354230), taken from storage at −20°C, were thawed overnight at 4°C to prepare for tubuloid culture plating. All culture plates were incubated overnight at 37°C prior to plating to aid in-Matrigel attachment and polymerization. For imaging applications, a 24-well glass bottom plate (Grenier Bio-One #662892) was used to set up cultures by first plating 150 μl Matrigel to cover the surface of the plate. The bottom layer was allowed to polymerize for 1–2 h at 37°C. During the polymerization step, PKD tubuloid medium was prepared. PKD tubuloid medium was made from 37 ml of Basic tubuloid medium [490 ml DMEM/F12, 1% penicillin-streptomycin (Sigma P4333), 1% ITS (Gibco 51500-056)] filtered with a 20 μm Steriflip, 5 ml fetal bovine serum (Sigma F0926), and a growth factor cocktail of 40 ng/ml recombinant human hepatocyte growth factor (HGF; Invitrogen, PHG0324), 20 ng/ml recombinant human epidermal growth factor (EGF; Sigma, E9644), and 8.8 ng/ml recombinant human basic fibroblast growth factor (FGF; Sigma, FO291). Mouse glial-derived neurotrophic factor (GDNF; 20 ng/ml; Sigma, SRP3200) was added to PKD tubuloid medium to make pulse medium. Following polymerization, frozen aliquots of primary tubule preparations (−80°C) were thawed and resuspended in prewarmed Basic tubuloid medium in a 15 ml conical tube coated with 2.64% BSA in 1×d-PBS. From this point forward, all plasticware, including tubes and pipette tips, was coated in 2.64% BSA solution. Cell suspensions were centrifuged for 3 min at 500 ***g*** and supernatant aspirated to remove residual DMSO from freezing medium. The cell pellet was resuspended in 400 μl of PKD tubuloid medium with GDNF and 100 μl of cell suspension was plated on top of the first polymerized Matrigel layer (4 wells per aliquot). Tubule preparations were allowed to attach and settle on Matrigel by incubating at 37°C for 1–2 h. Finally, another 150 μl of Matrigel basement membrane construct was added to cover the primary tubule preparations. This was the final layer of the sandwich plating technique. The top layer was allowed to polymerize and the cultures were covered with 1.5 ml PKD tubuloid medium with GDNF for 24–36 h. PKD tubuloid medium (without GDNF) was exchanged every other day by replacing 1 ml of medium each time throughout the culture period. Cultures were typically maintained for 10 days due to the longevity of Matrigel integrity.

### Inactivation of *Pkd2*, structure classification, and morphological tracking following *Pkd2* inactivation

Following a 3-day differentiation period after plating, tubuloids were treated with either 10 μg/ml of doxycycline (Sigma D3072) to inactivate *Pkd2*, or DMSO for controls, for 3 days. Following doxycycline treatment, individual structures and population tracking was continued every 24 h for up to 10 days. Individual structures were tracked by taking 20× objective magnification bright-field and fluorescence images to visualize changes in the mTmG reporter, while population tracking data was acquired as 4× objective magnification bright-field and fluorescence images. Positions for both levels of structure tracking were saved using Metamorph MultiDimensional Acquisition software [7.8.12.0 (August 27, 2015)] on an Olympus IX83 epifluorescence microscope with an ASI motorized stage. Classification of tubuloid structures was performed manually with defined description criteria for both differentiated and undifferentiated structural classifications (Fig. 2A). These classifications were determined using staining patterns of epithelial markers, including E-cadherin (eBioscience, 13-3249) and zonula occludens-1 (Z0-1; SCBT, sc-33725). The differentiated class of structures includes spheroids and tubules, which both stained positively for E-cadherin and ZO-1 (Figs 2B, 3D). Spheroids were defined by a multicellular wall with a clear lumen, whereas tubules exhibited a slit-like lumen with a diameter of about 50 μm. The undifferentiated structures were negative for the epithelial markers E-cadherin and ZO-1, which suggests a role as supportive, globular structures, called prestructures. For tubuloid analysis, structures were categorized according to the parameters in Fig. 2 from bright-field images taken with a 4× objective. This determination of tubule versus spheroid population was manually characterized through blinded categorization, and structures that were out of the plane of focus or not within the boundaries of the field of view were not included.

### Quantification of mTmG reporter fluorescence and protein abundance following Cre activation

Changes in fluorescence were quantified following 3 days of 10 μg/ml doxycycline treatment. Quantification of relative +mTmG fluorescence following activation of Cre recombinase was analyzed in ImageJ using the Color Pixel Counter Plugin [ImageJ v.1.0: initial release (5/5/2010)]. To validate protein changes in the double fluorescent reporter, fluorescence activated cell sorting was used to look at changes in PC2 in isolated red and green populations. Inducible cells were cultured on 10-cm dishes with REC medium (described above) and treated with doxycycline following the protocol for organoids. Cells were detached from the plate and prepared for sorting by the University of Maryland Greenebaum Comprehensive Cancer Center Flow Cytometry Shared Services. Cells were sorted using a BD Aria II Cell Sorter into GFP-positive (488 nm) and Tomato-positive (552 nm) populations. For immunoblotting analysis in 3D culture, tubuloids were recovered from the Matrigel using Corning Cell Recovery solution (VWR 354253) following the manufacturer's protocol. After removal of recovery solution, tubuloids were lysed with RIPA buffer containing deoxycholic acid (1% deoxycholic acid, 1% Triton X-100, 0.1% SDS, 150 mM NaCl, 1 mM EDTA, and 10 mM Tris-HCl pH 7.5) with 1:10 protease inhibitor (Sigma P-8340). Tubuloid lysate was rotated in the cold room at 4°C for 30 min and then centrifuged for 15 min at 16,000 ***g***. Supernatant was collected and protein abundance was quantified by BCA assay (Thermo Scientific 23225); pellets were saved and stored at −80°C. Samples were heated with 5× Laemmli buffer containing SDS and 10% β-mercaptoethanol for 30 min at 37°C. Samples were then loaded on 10% stain-free gels (BioRad 4568033) with kaleidoscope marker (BioRad 161-0375) and run for 50 min at 200 V. Before transfer, gels were crosslinked using UV (BioRad ChemiDoc MP Imaging System) and imaged to quantify total loaded protein (LC) for normalization of protein abundance. Gels were then transferred onto 0.2 μm pore size nitrocellulose membranes (BioRad 1704158) using a semidry BioRad Trans-blot Turbo System. Membranes were blocked in 5% milk in 1×TBS-T and primary antibodies (1:1000 rabbit anti-PC2; Baltimore PKD Research and Clinical Core Center, 3374) were incubated overnight in 2.5% milk in 1×TBS-T at 4°C. Blots were washed three times in 1×TBS-T, then incubated with secondary antibody (1:5000 HRP-conjugated goat anti-rabbit; Jackson ImmunoResearch Laboratories, 111035144) in 2.5% milk in 1×TBS-T for 1 h, with rocking, at room temperature. Blots were washed again three times with 1×TBS-T and developed in SuperSignal West Pico chemiluminescent substrate (Thermo Scientific 34577). Blots were developed using a Biorad Chemidoc imaging machine and quantified using ImageLab (BioRad Version 6.0.1 build 34). Normalization was performed by calculating the total protein loaded in each lane using the BioRad Stain-Free Gel System. Statistical comparisons of density measurements from western blots were performed using a Student's *t*-test for pairwise comparisons (Prism 7, GraphPad, USA). All reported data are means±s.e.m.

### Fixation

Following ∼10 days of culture and tracking, tubuloids were fixed in Matrigel basement membrane. First, medium was gently removed and the culture was washed with prewarmed 1×PBS, three times. Following removal of PBS wash buffer, wells were treated with 1:20 collagenase A (0.1 g/ml; Sigma C2139) and incubated at 37°C for 10 min to break down the Matrigel and improve penetration of fixative. The enzyme solution was removed and samples gently washed again with 1×PBS. Tubuloids were fixed in Matrigel with 3% paraformaldehyde (Electron Microscopy Sciences 15714-S) in 1×PBS on a rocking plate at room temperature for 30 min. Fixative was removed and samples were washed three times with 1×d-PBS. The plate was stored overnight at 4°C in 1×PBS following the fixation steps.

### Immunofluorescence

Following overnight storage of the plate, it was brought to room temperature and permeabilized by adding 0.025% saponin (Sigma S4521) in 1×PBS, rocking for 30 min at room temperature. The 1×PBS washes were repeated and samples were then blocked with 1% fish skin gelatin (Sigma G7765) in dH_2_O for 2 h at room temperature. The primary antibodies (rat anti-ZO-1, 1:50, sc-33725, SCBT; biotinylated E-cadherin, 1:200, 13-3249, Affymetrix; mouse anti-Na^+^/K^+^-ATPase, 1:200, 05-369, Millipore; mouse anti-acetyl-α-tubulin, 1:200, 12152S, CST) were added in the fish skin gelatin solution, rocking overnight at 4°C. The following day, the plate was allowed to re-equilibrate to room temperature to reduce the loss of structures during washing. The cultures were washed three times with room temperature 1×PBS. The fluorescent secondary antibody (goat anti-mouse Alexa Fluor 488, 1:200, A11001, Invitrogen; goat anti-rat Alexa Fluor 647, 1:200, A21247, Invitrogen; goat anti-mouse Alexa Fluor 555, 1:200, A21422, Invitrogen; streptavidin-conjugated Cy5, 1:25, 43-4316, Invitrogen) was added in 1% fish skin gelatin in dH_2_O and incubated while rocking at room temperature for 2 h. For DBA staining, 1:200 of rhodamine-conjugated DBA (Vector Laboratories RL-1032) was added in 1% fish skin gelatin with other secondary antibodies. The culture was washed again three times with 1×PBS and the sample was covered with mounting medium (Vector Labs H1200). Fixed culture plates were imaged on an Olympus IX83 inverted imaging system.

### Morphometric analysis and spherical agreement of structures before and after *Pkd2* inactivation

In a bright-field image (taken using a 20× objective magnification), a region of interest was drawn around each structure. The width, height, and area were recorded for each structure at timepoints before and 168 h after a 3 day doxycycline or DMSO treatment. Structures were quantified using the MetaMorph application for Region Statistics. Then, the spherical-like area was calculated (area=2π*r*^2^), using the shortest measurement of the width or height as the radius (*r*). The percentage difference between the area and calculated/spherical area was then compared before and after the addition of DMSO or doxycycline. The absolute value of this difference was graphed on a scale from 1 (spheroid) to 0 (tubule) to determine spherical agreement, or likeness to a sphere. A paired *t*-test was employed to compare spherical agreement before and after treatment on the same structure in DMSO or doxycycline conditions.

### RNA preparation

Tubuloids were isolated from the Matrigel using Corning recovery solution by following the manufacturer's protocol. For GDNF pulse experiments, tubuloids were collected 6 days following GDNF pulse on culture day 7 (Fig. 3). For *Pkd2* inactivation experiments, tubuloids were collected 2 days following the doxycycline or DMSO treatments on culture day 7 (Fig. 6). Once the pellet of organoids was isolated from the Matrigel, an RNeasy Plus Mini Kit (Qiagen 74134) was used to isolate RNA, following the manufacturer's protocol. RNA was suspended in molecular biology grade water and then quantified using a CLARIOstar plate reader before being stored at −80°C.

### Illumina sequencing

Libraries were prepared using the manufacturer's protocol for the Clontech SMART-Seq v4 Ultra Low Input RNA kit (minimum concentration 2 ng/μl; with poly-A selection). Samples were sequenced on two flow cell lanes of an Illumina HiSeq4000 75 bp paired end run. Four samples were sequenced in each flow cell lane. Sample quality assessment, RNA-Seq library preparation, and sequencing were performed by the Genomics Resource Center at the University of Maryland School of Medicine. RNA-Seq data was stored and analyzed on BasePair (https://app.basepairtech.com/) for expression count (STAR) and differential expression (DESeq2) analyses.

### Reverse transcription quantitative PCR

Following RNA isolation, cDNA was made using the SuperScript III First-Strand Synthesis system for RT-PCR (Invitrogen 18080-051) by following the manufacturer's protocol for first strand cDNA synthesis. Reactions (4 ng cDNA) were run in 96-well plates (Life Technologies 4483485) following the manufacturer's protocol for PowerUp SYBR Green (Life Technologies A25742). Oligonucleotides used were as follows: *Six2* forward primer, 5′-GAGGCCAAGGAAAGGGAGAA-3′; *Six2* reverse primer, 5′-GAACTGCCTAGCACCGACTT-3′; *Wnt11* forward primer, 5′-TCATGGGGGCCAAGTTTTCC-3′; *Wnt11* reverse primer, 5′-TTCCAGGGAGGCACGTAGAG-3′; *GATA3* forward primer, 5′-GCCCCTCATTAAGCCCAA-3′; *GATA3* reverse primer, 5′-GGAGGGAGAGAGGAATCCGA-3′.

### Visualizing GO terms using REViGO

Gene ontology (GO) terms that had a nominal *P*-value of less than or equal to 0.05 were added to a list on the REViGO webpage (http://revigo.irb.hr/) (Supek et al., 2011). Visualization criteria allowed for medium similarity (0.7), associated by *P*-values, selected to a *Mus musculus* GO database, using SimRel semantic similarity measurements.

### Visualizing differentially expressed genes using Heatmapper

Differentially expressed genes (adjusted *P*-value of less than or equal to 0.05 and fold change of 1.5) with their corresponding total number of reads per sample (raw read counts per gene divided by each sample's library size) were exported to a Microsoft Excel spreadsheet and uploaded into Heatmapper (http://www2.heatmapper.ca/expression/) (Babicki et al., 2016). Genes were mapped using average linkage and Pearson distance measurements.

### Human kidney protein sample preparation and immunoblotting

Normal (*n*=4) and ADPKD (*n*=5) human male kidney tissue samples were dissected from nephrectomized human kidneys received from the Baltimore PKD Research and Clinical Core Center and stored after flash freezing at −80°C (reviewed by the UMB IRB and determined to not be human research, requiring no further IRB review). Kidney tissue was homogenized using a BeadBug™6 homogenizer (Benchtop Scientific) with 3.0 mm beads (Benchmark Scientific, #D1032-30) in deoxycholic RIPA lysis buffer. Immunoblotting and development of human sample blots was performed as detailed above for tubuloid samples using a primary antibody against tensin-1 (1:100; Invitrogen, PA557515).

### Statistics

For RT-qPCR, fold changes (calculated using the ΔΔCt method) between experimental groups were compared using a two-tailed Student's *t*-test. For differentiation quantification, the percentage of differentiated structures or tubules of total structures was compared between experimental groups using a two-tailed Student's *t*-test. For analysis of western blots, specific protein abundance normalized to total loaded protein between experimental groups was compared using a two-tailed Student's *t*-test. For the spherical agreement assay, the change in morphometry of structures before and after a treatment were compared using a two-tailed paired sample *t*-test. RNA-Seq data was analyzed on BasePair (https://app.basepairtech.com/) for expression count (STAR) and differential expression (DESeq2) analyses (Love et al., 2014). Gene ontology terms were statistically compared using nominal *P*-values. Differentially expressed genes were statistically compared using adjusted *P*-values calculated with a Benjamini-Hochberg adjustment using an alpha value of 0.1.

## Supplementary Material

Supplementary information
