# Supplementary material for: GDNF drives rapid tubule morphogenesis in a novel 3D in vitro model for ADPKD
Source: J Cell Sci. 2020 Jul 16;133(14):jcs249557. doi: 10.1242/jcs.249557 (PMC7375472; doi:10.1242/jcs.249557)
Supplement: Supplementary information [file joces-133-249557-s1.pdf]

Fig. S1: Tubuloid culture with *Pkd1fl/fl Pax8rtTA TetOCre +mTmG*

A

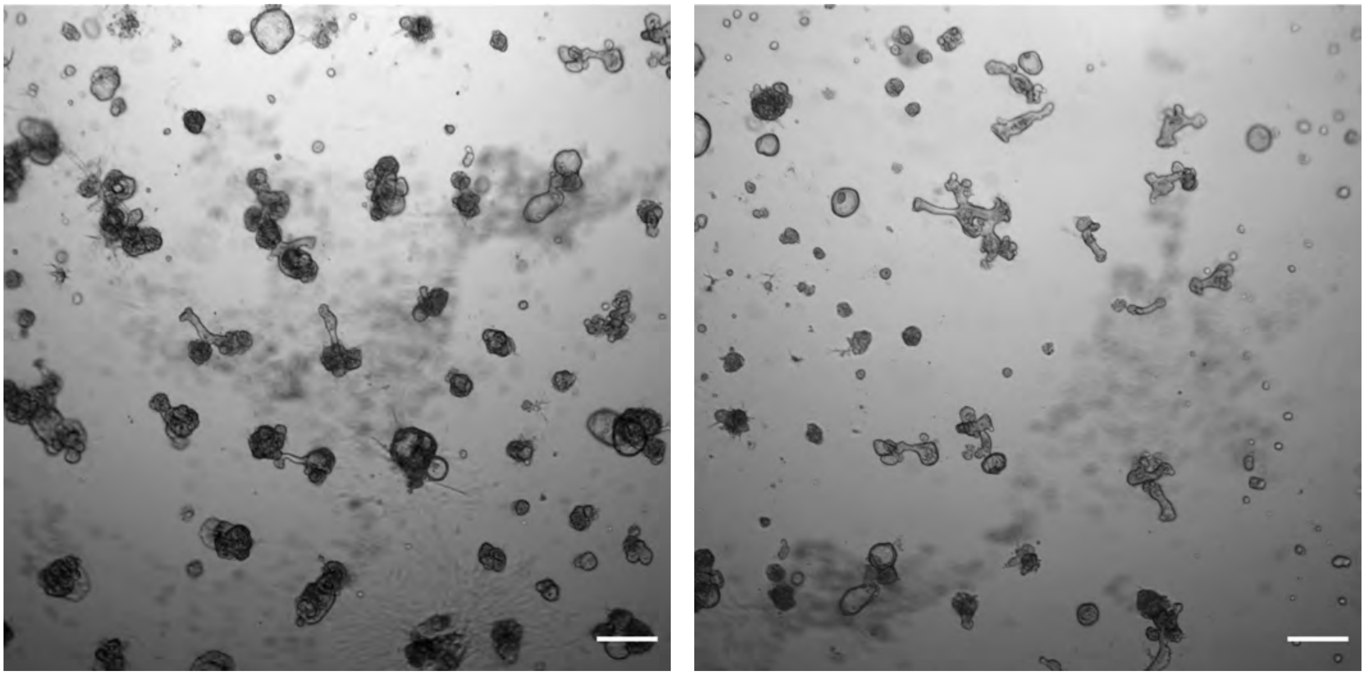

(A) Culture protocol can implement primary cells from mouse models with other genotypes. Brightfield images of tubuloids from *Pkd1fl/fl Pax8rtTA TetOCre +mTmG*. Magnification 4X, Scale Bar 250 µm.

Journal of Cell Science • Supplementary information

Fig. S2: Western blots and total loading controls for PC2

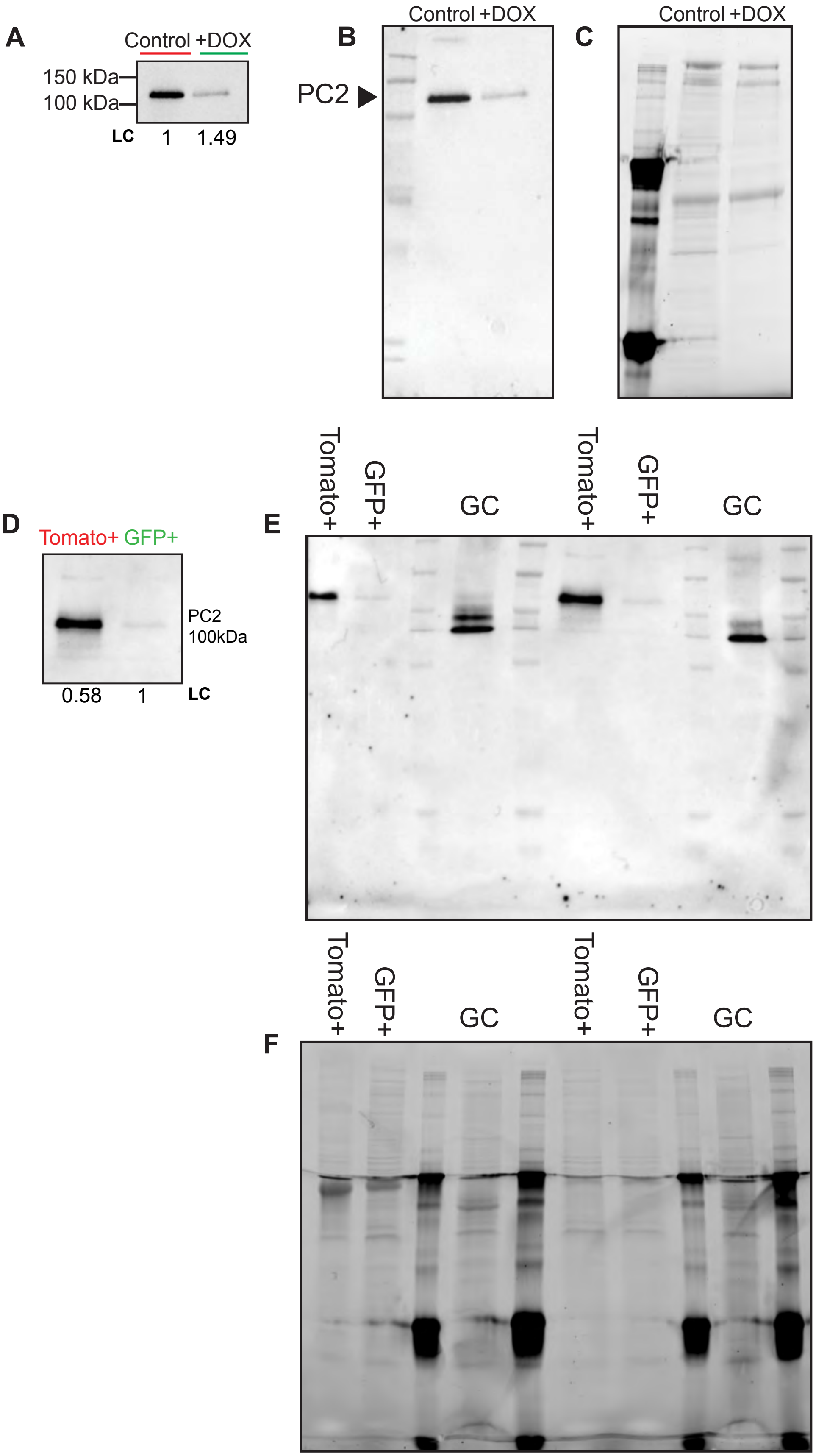

(A) Western blot for PC2 abundance, normalized to total loaded protein (LC) (n=3; two-tailed Student's t-test, p-value = 0.0420). (B) Uncropped representative western blot for PC2 in organoids as seen in Figure 4C. (C) Total loaded protein used for normalization of blot in (A).(D) Western blot to confirm significant decrease in PC2 abundance in green fluorescing cells (GFP+) when compared to controls (Tomato+), normalized to total loaded protein (LC) (two-tailed Student's t-test, p-value = 0.0336). (E) Uncropped representative western blot for PC2 in FACS cells as seen in Figure 4D, with gating control (GC). (F) Total loaded protein used for normalization of blot in (D).

Fig. S3: Expression of proliferation genes in *Pkd2-* tubuloids

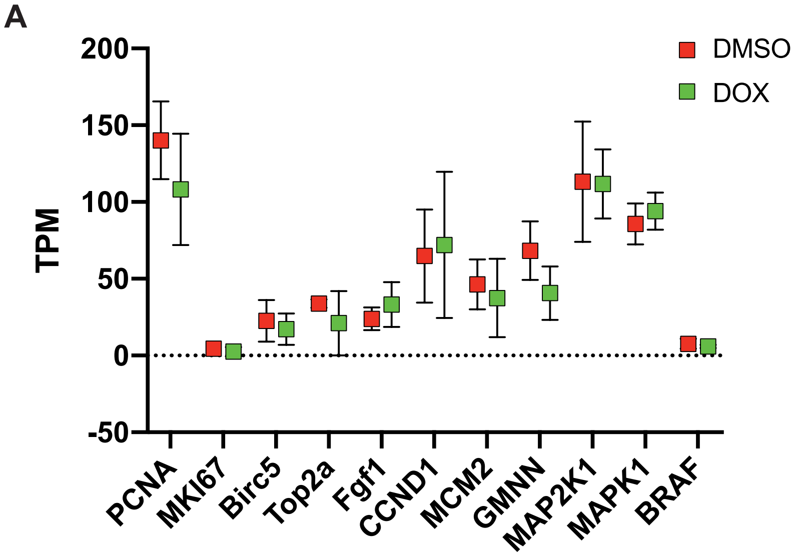

(A) A profile of ten proliferation genes reveals no signficant change in expression between doxycycline treated (green square) and DMSO control (red square) tubuloids (n=4). Signficance was defined in a two-tailed Student's t-test as a p-value of less than or equal to 0.05. Bars represent +/-SEM and those not seen are hidden behind boxes.

Fig. S4: Western blots and total loading controls for tensin-1

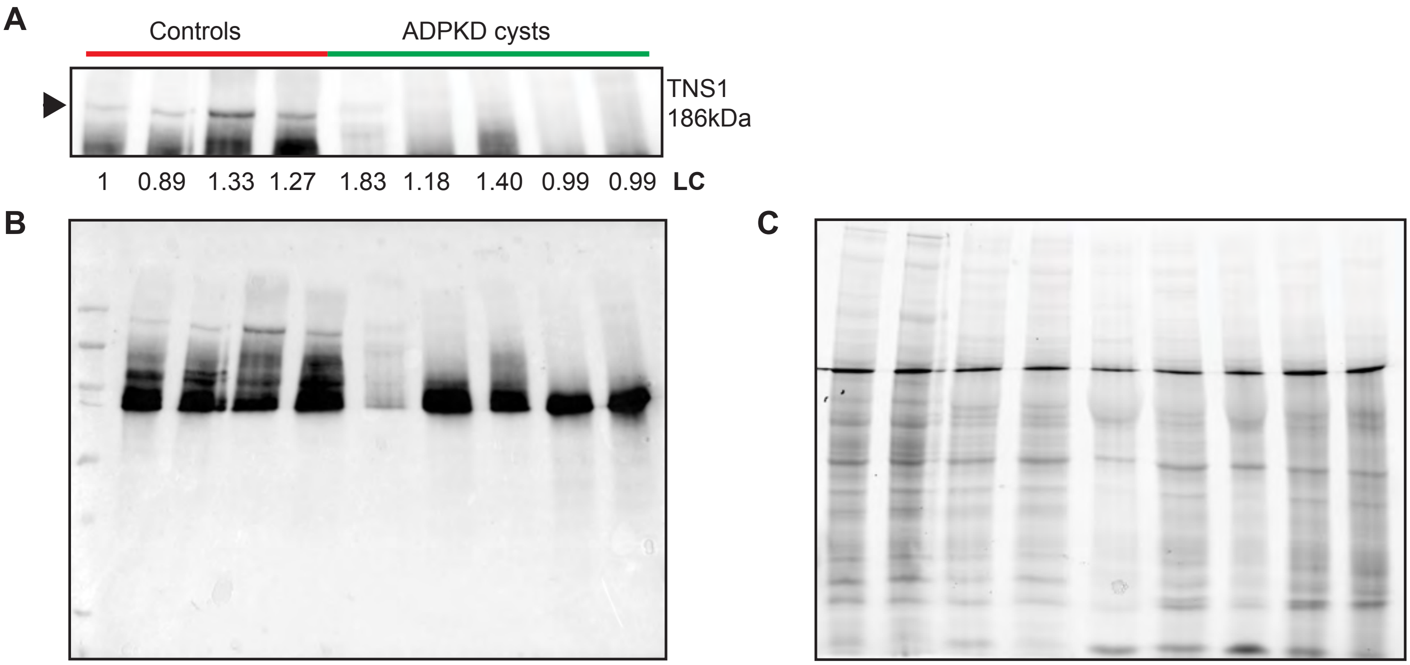

(A) Western blot exhibits significant decrease in tensin-1 (TNS1) in human male ADPKD cysts (n=5) compared to normal human male kidneys (n=4) and normalized to total loaded protein (LC) (two-tailed Student's t-test, p-value = 0.0340). (B) Uncropped western blot for TNS1 in human tissue as seen in Figure 6D. (C) Total loaded protein used for normalization of blot in (A).

Table S1: GDNF pulse differentially expressed genes

A list of genes with reported fold change, p-value, and adjusted p-value (≤0.05) resulting from differential expression (DESeq) analysis of GDNF pulsed (n=4 cultures) and control (n=4 cultures) tubuloids.

Click here to Download Table S1

Table S2: GDNF pulse differential pathway analysis

A list of significantly altered gene ontology (GO) terms from pathway analysis of GDNF pulsed (n=4 cultures) and control (n=4 cultures), determined by nominal p-value (≤0.05) along with reported values for size, es, nes, fdrQVal, fwerPVal, and rankAtMax. From significantly altered GO terms, non-neuro specific GO terms and MGI GO number with nominal p-value were included in REViGO representation.

Click here to Download Table S2

Table S3: GDNF pulse PC TF analysis

GENE	GDNF1	GDNF2	GDNF3	GDNF4	CTL1	CTL2	CTL3	CTL4	One-tailed Student's t-test
Gata3	32.26604	15.72488	35.3853	40.9055	24.96285	9.642504	24.51733	13.17762	0.049949809
Tfap2b	3.052619	1.859329	4.514373	7.2772	2.650732	0.593037	3.272445	3.129981	0.115260295
Ehf	33.49256	13.52809	37.78405	40.72878	21.69552	5.139788	22.66092	17.01686	0.04549041
Irf1	41.69357	27.52123	43.46952	37.97642	31.63945	24.41116	29.73884	19.68488	0.022550114
Irf9	24.43075	14.76461	23.20745	22.94073	13.58074	14.81047	16.50369	10.28917	0.013168292
Stat1	10.12961	7.084977	6.76742	9.216172	7.086282	5.745113	5.906681	4.200367	0.021996706
Tsc22d3	22.12605	18.84984	17.24039	13.46117	14.82216	29.72123	17.12784	17.69298	0.315742822
Nfia	9.771741	8.088848	6.936182	5.848866	4.677708	6.133078	4.613625	1.950422	0.016812074
Maf	9.315971	7.586065	9.245634	7.061692	5.883636	4.943121	7.791981	2.774088	0.023882416
Nfix	8.523388	7.49054	10.18001	8.829175	6.379807	6.788277	7.339321	3.597376	0.017148745
Glis2	23.42146	20.22029	36.06616	29.29902	18.2061	14.47908	20.82394	10.46062	0.01755182
Grhl2	13.71164	7.388358	15.9763	11.19042	11.66951	1.548885	9.833759	6.625391	0.078952635

Table S3: GDNF pulse PC TF analysis

Transcripts per million (TPM) are reported for each of the 12 principal cell specific transcription factors (Fig. 3) from GDNF pulsed (n=4 cultures) and control (n=4 cultures) tubuloids with p-values, determined by one-tailed Student's t-test.

Journal of Cell Science • Supplementary information

Table S4: Proliferation genes in *Pkd2-* tubuloids

GENE	DOX1	DOX2	DOX3	DOX4	DMSO1	DMSO2	DMSO3	DMSO4	Two-tailed Student's t-test
Pcna	132.0346	72.45922	146.0151	82.279	120.8519	121.7592	174.7354	143.3277	0.198243095
Mki67	1.84064	0.514181	6.985879	0.887624	2.314329	4.406596	7.160676	4.035646	0.328269372
Birc5	15.05231	14.21884	31.59758	7.910873	3.513497	32.02806	32.26265	22.77518	0.542083124
Top2a	17.04601	2.512155	50.96887	13.71334	34.49851	30.01402	34.47899	36.5376	0.269189562
Fgf1	28.08196	54.17641	30.51607	20.29025	24.06256	13.53972	29.65386	28.47016	0.296875262
Ccnd1	27.52501	34.37395	110.4346	115.9078	51.53557	34.25258	105.1313	68.45452	0.806612205
Mcm2	32.82517	5.989377	67.66323	43.3237	70.25122	34.48768	38.81866	41.99352	0.576219144
Gmnn	32.78538	51.02927	58.67824	20.23719	63.26325	63.86299	95.36338	50.55107	0.076769
Map2k1	90.28978	103.3186	110.5618	143.1013	100.5623	153.0988	64.42082	134.8364	0.952167396
Mapk1	90.18806	79.34079	107.6615	99.08689	104.5303	76.477	75.97767	85.87185	0.389986305
Braf	5.33684	5.976084	7.261508	4.97417	11.38655	5.905678	9.284145	4.080653	0.341010592

Table S4: Proliferation genes in *Pkd2-* tubuloids
Transcripts per million (TPM) are reported for each of the 11 proliferation genes from DOX treated (*Pkd2-)* (n=4 cultures) and DMSO treated (n=4 cultures) tubuloids with p-values, determined by two-tailed Student's t-test.

Journal of Cell Science • Supplementary information

Table S5: *Pkd2-* differentially expressed genes

GENE	Key	Fold Change (log2)	P-value	P-adj
Tns1	21817	-0.886637	1.54E-09	2.13E-05
Fhod3	7436	-2.2331063	6.24E-08	0.000246
Plscr2	16979	-0.551824	4.80E-08	0.000246
Rp2h	18538	-0.7319563	7.13E-08	0.000246
Ttc7b	22225	0.96903559	1.01E-07	0.000278
Lurap1l	11828	-0.8584363	2.04E-07	0.000469
Cyp11a1	5398	-3.95454	2.50E-07	0.000493
Uap1l1	22345	1.07427609	5.74E-07	0.000991
Cnpy3	4867	0.74646905	7.22E-07	0.001109
Renbp	18194	0.9932179	1.81E-06	0.002502
Cdsn	4356	4.45575235	5.54E-06	0.006956
Grhl3	9482	5.2921039	1.05E-05	0.012112
Zhx2	23940	0.70640915	1.35E-05	0.014314
Ankrd37	2440	-3.1662621	1.48E-05	0.014521
Hdhd2	9818	0.34203803	1.58E-05	0.014521
B630005N14Rik (Bmt2)	3116	-0.8049442	1.84E-05	0.015883
Heatr2	9823	0.70116212	2.06E-05	0.016785
Alg6	2292	-0.8873579	2.24E-05	0.017181
Eprs	6736	1.02868284	2.61E-05	0.018066
Slc3a2	19719	0.83796655	2.49E-05	0.018066
Nfkbiz	14381	-1.2770209	2.76E-05	0.018139
Tph1	21873	-5.1697975	3.05E-05	0.019149
Psen2	17578	1.45523883	3.7E-05	0.02223
Lars	11396	0.94356128	5.13E-05	0.029549
Cox7a1	5027	-3.5191817	5.72E-05	0.030392
Slc1a3	19515	2.13120498	5.66E-05	0.030392
Krt5	11219	9.00795606	7.85E-05	0.040196
Ccnd2	4084	0.63569798	9.14E-05	0.042103
Cd38	4188	1.03533186	9.09E-05	0.042103
Mapkap1	12046	0.53194512	9.02E-05	0.042103
Slc6a9	19790	1.57457384	9.58E-05	0.042698
Adamts5	2030	-1.623841	0.00011	0.047624
Gaa	7711	0.88069113	0.000117	0.047624
Mfsd7c	12332	1.89261777	0.000117	0.047624
Etv1	6850	0.83819896	0.000123	0.048598

Table S5: *Pkd2-* differentially expressed genes
A list of genes with reported fold change, p-value, and adjusted p-value (≤0.05) resulting from differential expression (DESeq) analysis of DOX treated (*Pkd2-*) (n=4 cultures) and DMSO treated (n=4 cultures) tubuloids, represented in volcano plots and Heatmapper in Fig. 6.

Journal of Cell Science • Supplementary information

Table S6: Annotated *Pkd2* - differentially expressed genes

Down- and upregulated differentially expressed genes in *Pkd2* - (DOX treated) tubuloids annotated with protein, function, and corresponding fold change. Functions were summarized described from National Library of Medicine (US), National Center for Biotechnology Information.

GENE (DOWN)	Protein	Function [Human]	Fold Change (log2)
Tph1	Tryptophan hydroxylase 1	encodes protein that catalyzes the rate-limiting step of serotonin biosynthesis	-5.1697975
Cyp11a1	Cholesterol side-chain cleavage enzyme, mitochondrial	encodes protein that localizes to the mitochondrial inner membrane and catalyzes the conversion of cholesterol to pregnenolone, the rate-limiting step in the synthesis of the steroid hormones	-3.95454
Cox7a1	Cytochrome c oxidase subunit 7A1, mitochondrial	encodes component of heteromeric complex involved in electron transfer	-3.5191817
Ankrd37	Ankyrin repeat domain 37	undefined	-3.1662621
Fhod3	FH1/FH2 domain-containing protein 3	encodes protein that is part of the diaphonous-related formins and has a predicted role in actin filament polymerization	-2.2331063
Adamts5	A disintegrin and metalloproteinase with thrombospondin motifs 5	encodes a protein in the ADAMTS family that is involved in proteoglycan cleavage	-1.623841
Nfkbiz	NF-kappa-B inhibitor zeta	encodes a member of the ankyrin-repeat family and is involved in inflammatory responses	-1.2770209
Alg6	Alpha-1,3-glucosyltransferase	encodes a protein of the ALG6/8 glucosyltransferase family that catalyzes the addition of glucose residue for N-linked glycosylation	-0.8873579
Tns1	Tensin-1	encodes a protein that localizes to the focal adhesions and crosslinks with the actin cytoskeleton; involved in signal transduction	-0.886637
Lurap1l	Leucine rich adaptor protein 1-like	undefined	-0.8584363
B630005N14Rik (Bmt2)	base methyltransferase of 25S rRNA 2	undefined	-0.8049442
Rp2h	retinitis pigmentosa 2 homolog or RP2 activator of ARL3 GTPase	predicted gene product may be involved in beta-tubulin folding	-0.7319563
Plscr2	Phospholipid scramblase 2	encodes a membrane protein that mediates plasma membrane exposure to phospholipids and phosphatidylserines	-0.551824

Journal of Cell Science • Supplementary information

Down- and upregulated differentially expressed genes in *Pkd2*- (DOX treated) tubuloids annotated with protein, function, and corresponding fold change. Functions were summarized described from National Library of Medicine (US), National Center for Biotechnology Information.

GENE (UP)	Protein	Function [Human]	Fold Change (log2)
Krt5	Keratin 5	encodes a member of the keratin gene family coexpressed during differentiation of epitheilial tissues	9.00795606
Grhl3	Grainyhead-like transcription factor 3	encodes for a protein that may act as a transcription factor during development to stimulate cell migration	5.2921039
Cdsn	Corneodesmosin	encodes a protein involved in corneocyte maturation and localizes to cornified epithelia and epidermis	4.45575235
Slc1a3	Solute carrier family 1 member 3	encodes a high affinity glutamate transporter	2.13120498
Mfsd7c	FLVCR heme transporter 2	encodes a transmembrane calcium transporter that may be involved in development	1.89261777
Slc6a9	solute carrier family 6 member 9	encodes a transporter involved in inhibition of glycine signaling	1.57457384
Psen2	Presenilin-2	encodes a component of multi-subunit gamma secretase complex that play roles in cellular processes and calcium homeostasis	1.45523883
Uap1l1	UDP-N-acetylglucosamine pyrophosphorylase 1 like 1	undefined	1.07427609
Cd38	CD38 molecule	encodes a transmembrane glycoprotein that synthesizes an intracellular calcium ion mobilizing messenger	1.03533186
Eprs	glutamyl-prolyl-tRNA synthetase	encodes a multifunctional aminoacyl-tRNA synthetase that catalyzes aminoacylation of glutamic acid and proline tRNA species	1.02868284
Renbp	Renin binding protein	encodes a product that inhibits renin and is a GlcNAc 2-epimerase	0.9932179
Ttc7b	Tetratricopeptide repeat protein 7B	undefined	0.96903559
Lars	Leucyl-tRNA synthetase	encodes a cytosolic leucine-tRNA synthetase that catalyzes the ATP-dependent ligation of L-leucine to tRNA	0.94356128
Gaa	Glucosidase alpha, acid	encodes lysosomal alpha-glucosidase	0.88069113

Down- and upregulated differentially expressed genes in *Pkd2*- (DOX treated) tubuloids annotated with protein, function, and corresponding fold change. Functions were summarized described from National Library of Medicine (US), National Center for Biotechnology Information.

GENE (UP)	Protein	Function [Human]	Fold Change (log2)
Etv1	ETS variant transcription factor 1	encodes a protein that plays a role in cell growth, angiogenesis, migration, proliferation, and differentiation	0.83819896
Slc3a2	Solute carrier family 3 member 2	encodes a transporter that regulates intracellular calcium levels and transports L-type amino acids	0.83796655
Cnpy3	Canopy FGF signaling regulator 3	encodes a protein that aids in toll-like receptor maturation and acts as a chaperone for protein export	0.74646905
Zhx2	Zinc fingers and homeoboxes 2	encodes for nuclear homodimeric transcriptional repressors that interact with the A subunit of nuclear factor-Y	0.70640915
Heatr2	Dynein axonemal assembly factor 5	encodes a protein needed for axonemal dynein arms in organisms with motile cilia and flagella	0.70116212
Ccnd2	Cyclin D2	encodes a protein in the cyclin family and functions as a regulator of CDK kinases	0.63569798
Mapkap1	Mitogen-activated protein kinase associated protein 1	encodes a protein similar to stress activated protein kinases	0.53194512
Hdhd2	Haloacid dehalogenase-like hydrolase domain containing 2	undefined	0.34203803

Journal of Cell Science • Supplementary information
